# Exploration capacity versus specific enzymatic activity of ectomycorrhizas in response to primary productivity and soil phosphorus availability in Bornean tropical rainforests

**DOI:** 10.1038/s41598-024-53234-6

**Published:** 2024-02-03

**Authors:** Kei-ichi Okada, Daiki Yokoyama, Shin-ichiro Aiba, Kanehiro Kitayama

**Affiliations:** 1 Faculty of Bioindustry, Tokyo University of Agriculture, Abashiri, Japan; 2 Graduate School of Environment and Information Sciences, Yokohama National University, Yokohama, Japan; 3 Graduate School of Agriculture, Kyoto University, Kyoto, Japan; 4 Center for Sustainable Resource Science, RIKEN, Yokohama, Japan; 5 Faculty of Environmental Earth Science, Hokkaido University, Sapporo, Japan

**Keywords:** Ecology, Ecosystem ecology, Forest ecology, Microbial ecology, Tropical ecology

## Abstract

Ectomycorrhizal (ECM) fungi are functionally important in biogeochemical cycles in tropical ecosystems. Extracellular enzymatic activity of ECM on a ground-area basis is the product of two attributes; exploration capacity (ECM surface-area) and specific enzymatic activity. Here, we elucidated which attribute better explained the ECM enzymatic activity in response to different levels of soil phosphorus (P) and Nitrogen (N) availability in five Bornean tropical rainforests. We determined the surface area of ECM root tips as well as the enzymatic activities per ECM surface area for carbon (C), N and P degrading enzymes in each site. We evaluated the relationship of ECM enzyme activities with the resource availabilities of C (Above-ground net primary production; ANPP), N, and P of ECM by a generalized linear mixed model. The ECM enzymatic activities on a ground-area basis were more significantly determined by specific enzymatic activity than by the exploration capacity. Specific enzymatic activities were generally negatively affected by C (ANPP) and soil P availability. ECM fungi enhance the specific enzyme activity rather than the exploration capacity to maintain the capacity of nutrient acquisition. The less dependence of ECM fungi on the exploration capacity in these forests may be related to the limitation of C supply from host trees. We highlighted the adaptive mechanisms of ECM fungi on nutrient acquisition in tropical ecosystems through the response of enzymatic activity to nutrient availability across the elements.

## Introduction

Tropical ecosystems exhibit the highest net primary productivity of all terrestrial ecosystems, which plays a crucial role in the global carbon (C) cycle. At the same time, primary productivity in tropical ecosystems is often limited by insufficient soil phosphorus (P) availability because of the high weathering rates of the soil minerals and an associated geochemical transformation of soil P into unavailable forms^1–4^. In order to address the mechanisms of how tropical trees maintain primary productivity on such soils with reduced P availability, many studies have focussed on the ecophysiology of the efficient P-use in photosynthetic C assimilation^1,5–7^, and on the nutrient acquisition strategy of plant root systems^8,9^. However, the roles of root-associated microorganisms on biogeochemical P cycles and the plant P acquisition strategy have not fully been understood yet.

Most terrestrial plants rely on mycorrhizal fungi associated with plant roots for mineral nutrient acquisition. Mycorrhizal fungi can take up both mineral and organic forms (amino acid) of nutrients from soils by expanding mycorrhizal root tips and extraradical hyphae, both of which function to enhance exploration capacity^10,11^. More specifically, ECM have a larger nutrient foraging area, which benefits the host plant. Because ECM fungal sheath can often enclose the colonized root surface^12^, the direct contact of the fungal sheath with the soil act as a substitute for the enclosed root surface. ECM fungi have a significant physiological capacity to access nutrients bound with complex organic compounds including cellulose, protein, chitin, and phytate^12–15^. Some specialized species can also access recalcitrant organic matters such as lignin and phenol-complexes, and they have specific forms of extracellular enzymes to degrade such recalcitrant matter^16^. Extracellular enzymatic activities associated with the degradation and nutrient release from soil organic matter reflect the functional diversity of ECM fungal community in situ^17,18^. Substantial differences in enzymatic activities have been reported among ECM fungal species in a given site or across sites along environmental gradients^17,19–22^, This suggests that changes in ECM fungal communities and their host trees can modify ECM enzyme activities, adapting to nutrient availability variations across sites^23–25^. Despite these significant potential of ECM fungi on biogeochemical cycles, only a few measurements of extracellular enzymatic activities of ECM have been reported from tropical soils^26^. Moreover, the adaptive response of ECM fungi under nutrient deficiency has been poorly elucidated so far. This oversight is particularly critical when considering the vital role of ECM fungi in facilitating tree adaptation and survival in low-phosphorus (P) soils.

Soil microbes including ECM fungi secrete extracellular enzymes including those that mineralize C, N and P to access each nutrient. The relative activity of those enzymes is suggested to be controlled by the availability of reactive substrates and/or soil mineral nutrients (i.e., the product of the enzyme reaction of each nutrient element)^27^. For example, phosphatase activity of soil microbes and its ratio to the C mineralizing enzyme activity in tropical ecosystems were greater where soil organic-P fraction was relatively small^28,29^. Thus, the stoichiometry of given two enzymes (enzymatic stoichiometry; i.e. the ratio of the activities of two enzymes) can vary with relative substrate abundances and/or nutrient availability and reflects the enzyme allocation of soil microbes^28^.

In Southeast Asia, diverse ECM host trees occur as dominant taxa such as the families Dipterocarpaceae, Fagaceae and Myrtaceae^30–33^. The abundance of these taxa could be related to soil nutrient availability, depending on the nutrient acquisition strategy of each taxa through ECM symbioses. Mount Kinabalu, Borneo, is one of the hotspots of world floristic biodiversity and diverse species of ECM host trees occur within/across sites^34^. Soil nutrient availability of P (and N) is highly variable due to complex geology and a wide altitudinal gradient on this mountain^5,35–38^. ECM fungal communities also remarkably changed along these gradients^39^. This natural setting on Mount Kinabalu is ideal for investigating the adaptive responses of ECM fungi in terms of nutrient acquisition capacity.

Here, we aimed to elucidate the adaptive strategy of ECM symbiosis in the tropical ecosystems by investigating the response of the extracellular enzymatic activity of ECM fungi to contrasting soil P and N availability in five tropical rain forests on Mount Kinabalu. We evaluated the enzymatic activity of ECM from two aspects, contact area with soil as an exploration capacity (biomass and surface area of ECM on a ground-area basis) and specific enzymatic activity (enzymatic activity on an ECM surface-area basis). We also characterized the stoichiometric relationships among enzymatic activities degrading different organic elements (C, N, and P). Subsequently, we evaluated the realized ECM enzymatic activity on a ground-area basis by integrating the two aspects of ECM (exploration capacity and specific enzymatic activity) to clarify the overall performance of ECM symbiosis in response to P availability. We hypothesized that ECM fungi, as an adaptive response in P-deficient forests with limited productivity, enhance P acquisition by increasing both exploratory capacity and specific enzymatic activity, especially for the P-specific enzyme.

## Materials and methods

### Site description

Five tropical rain forests on Mount Kinabalu (4095 m a.s.l.; 6°5′ N, 116° 33′ E), Borneo, were selected for this study. These include two lowland forests and three montane forests based on the vegetation classification by Kitayama^35^. These study sites were part of the study plots for the ecosystem dynamics project designed by Aiba and Kitayama^34^ and Kitayama et al.^38^. The climate of the study sites is humid equatorial with little seasonality in air temperature and precipitation. The mountain is non-volcanic and largely consists of Tertiary sedimentary rocks of sandstone and/or mudstone and ultrabasic rocks that protruded the sedimentary rocks as mosaics. Two geological substrates (sedimentary and ultrabasic soils) were selected in each of the lowland zone (700 m a.s.l.) and lower montane zone (1700 m a.s.l.), yielding a total of four forests in a matrix manner of altitude and substrate. The fifth forest is located in the lower montane zone at 1700 m on Quaternary tilloid deposits mostly of sedimentary rocks (hereafter Quaternary substrate)^38^. All sites are on gentle slopes to avoid the effects of topography. The ecosystem properties of the sites can be referred to (Table 1)^5,34,38^.

**Table 1:** Description of five study sites on Mount Kinabalu, Borneo^5,36–38,46^.

Site		Symbol	Exact altitude (m)	MAT (°C)	MAP (mm)	Biomass		ANPP
						Above-ground	Fine-root	
						(kg m^−2^)		(g m^−2^ yr^−1^)
Lowland	Tertiary	07T	650	23.9	2509	40.5	0.139	2101
700 m a.s.l	Ultrabasic	07U	700	23.7	2509	67.7	0.226	1873
Montane	Tertiary	17T	1560	18.9	2714	29.4	0.161	1141
	Ultrabasic	17U	1860	17.3	2714	24.3	0.146	903
1700 a.s.l	Quaternary	17Q	1860	17.3	2714	31.8	0.228	1418

*MAT* mean annual air temperature, *MAP* mean annual precipitation, *ANPP* above-ground net primary productivity, *NUE* nitrogen-use efficiency, *PUE* phosphorus-use efficiency.

### Soil P availability

For soil P availability, the size of soluble inorganic P pools, extracted with hydrochloric-ammonium fluoride solution, varied substantially among the five forests due to differences in geology and weathering as a function of altitude^40^. It was always greater on sedimentary than on ultrabasic substrate at the same altitude (Table 2)^5,37^. Among the three forests at 1700 m, the pool size of soluble inorganic P was the greatest on the Quaternary substrate of the relatively young age reflecting the lesser weathering of soil minerals (Table 2)^38^.

**Table 2:** Soil chemical properties and soil P availability indices in five study sites on Mount Kinabalu, Borneo^5,29,36–38^.

Site	pH	C/N	Total N	N mineralization rate	Soluble-P	Labile Po
	(H_2_O)	ratio	(%)	(µg g^−1^ 10 days^−1^)	(g m^−2^)	(g m^−2^)
07T	4.1	13.8	0.21	19.9	0.18	2.23
07U	4.0	13.7	0.32	8.5	0.14	3.50
17T	4.5	11.4	0.21	− 0.01	0.14	4.71
17U	5.4	12.1	0.28	1.6	0.04	0.82
17Q	4.2	13.0	0.56	7.8	0.19	13.55

Abbreviations for sites: 07T, lowland forest on Tertiary sedimentary rocks; 07U, lowland forest on ultrabasic serpentine rocks; 17T, montane forest on Tertiary sedimentary rocks; 17U, montane forest on ultrabasic serpentine rocks; 17Q, montane forest on Quaternary sedimentary rocks. Po; Organic Phosphorus.

### Distribution of ectomycorrhizal host trees

The tree-species composition of the five forests was investigated by Aiba et al.^41^ and Aiba and Kitayama^34^. Three families of ECM host trees (Dipterocarpaceae, Fagaceae, and Myrtaceae) were distributed in all study sites. Only the genus *Tristaniposis* was regarded as an ectomycorrhizal host within the family Myrtaceae^31^. Indeed, we confirmed the ECM formation of *Tristaniopsis* by observing its ECM tips collected from seedlings in the study sites in our preliminary survey (Figure S1). No ECM tips were observed on the seedlings of *Leptospermum* or *Syzygium* (both Myrtaceae), which were also abundant in some of our sites. The relative basal area (RBA) of ECM host trees in each site was sorted by family based on the dataset (Table S1) ^34^. RBA of ectomycorrhizal host trees ranged from 21.4% at the Quarternary montane forest (17Q) to 38.5% at the ultrabasic lowland forest (07U).

### Sampling of ectomycorrhizal tips

ECM tips were collected twice for two measurements separately; biomass and enzyme activity assay of ECM tips. To measure the ECM biomass on a ground-area basis, ten soil cores (2 cm diameter, 15-cm depth) were collected at random positions (at least 10 m apart) from the surface layer including the A_0_, A, and E soil horizons in each study site in September 2012. Each soil core was kept in a plastic bag and stored at 4ºC for up to 2 weeks until further processing. Each soil sample was sieved (500 µm mesh) and rinsed with tap water to remove soil particles. The remaining fine roots (diameter < 2 mm) were then transferred to a Petri dish for stereomicroscopic observation. ECM tips were identified based on the presence of fungal mantle structure^42^, and active ECM tips with turgid surfaces were collected from fine roots. The ECM tips were scanned using a flat screen scanner (GT-X970, EPSON, Tokyo, Japan) and analyzed for tip surface area using Win-Rhizo (Regents Instruments Inc., Quebec, Canada). Scanned ECM tips and fine roots were dried for 3 days at 70ºC and weighed for determining dry mass. Consequently, the mean mass (g m^−2^) and surface area of ECM tips per unit ground-area (m^2^ ECM m^−2^), and ECM dry mass ratio to fine-root (w/w) were calculated for each site.

To determine specific enzyme activity of ECM, ECM tips were resampled close enough to host trees to trace their roots in the study sites in October 2014. We selected target species from dominant ECM host genera (Table S2). Three to seven mature ECM host trees were selected in each species in each site except one species of *Castanopsis* in the Tertiary lowland site where only one mature tree was found. The target host tree genera and the number of sampling trees are shown in Table S2 and S3, respectively. Three to five ECM root clusters were collected from each host tree by tracing the lateral roots from the base of a target tree. The total number of root cluster samples collected for each genus in each site ranges from 15 to 23 (Table S3). Root cluster samples were stored at 4 °C for up to 2 weeks until further processing^43^. Root clusters were cleaned as in the case of ECM biomass described above and were immediately applied to the assay of enzyme activity.

### Assay of ectomycorrhizal enzyme activity

More than twelve ECM tips (approximately 2 mm) of each root cluster were subjected to the measurements of potential specific ECM activity of five enzymes degrading organic C, N, and P as follows; β-glucosidase (BG, which hydrolyzes cellobiose into glucose), β-N-acetyl-glucosaminidase (NAG, which is related to chitin degradation), leucine-amino peptidase (LAP, which breaks down polypeptides), acid phosphatase (AP, which releases orthophosphate residues from phosphomonoesters) and polyphenol oxidase (PPO, which degrades lignin by oxidizing phenols). BG, NAG, LAP, and AP participate in labile organic matter decomposition, whereas PPO participate in recalcitrant organic matter decomposition. Eight non-ectomycorrhizal (NM) tips obtained from root clusters of each site were also added to the enzyme assay as control (i.e. to determine enzyme activities without ECM). A sequential assay of enzymatic activities in 96-well plates (AcroPrep 96-filter plate with 30–40 μm mesh size: Pall Life Sciences, Germany)^43^ was applied with some modification. The concentration of substrates and incubation volume were set following the optimized protocol^43^ except for PPO, for which the concentration of substrate L-DOPA and its incubation volume was set at 25 mM and 50 μl, respectively^44^. Incubation was conducted under a pH and temperature condition of the field in each site (Table 2). Fluorescence was measured at 364 nm excitation and 450 nm emission in the Corona Grating Microplate Reader SH-9000 (Corona Electric, Japan). The assay for PPO was measured spectrophotometrically at 460 nm. Assayed root tips were scanned and analyzed for root surface area as with the case of the ECM biomass described above. Enzyme activities were calculated from fluorimeter and photometer readings^43^. Because the determination of enzymatic activities relies on the rate of the cleavage of specific substrates by functional enzymes present at the surface of ECM root tips^42,43^, specific enzyme activity was expressed for each enzyme as the molar amount of released substrate per minute per unit ECM surface area (pmol min^−1^ mm^−2^). Specific enzyme activity was averaged in each root cluster for statistical analysis.

### Statistical analysis

The significant differences among study sites were compared by analysis of variance (ANOVA) followed by Tukey’s HSD post hoc test for the following variables: biomass on a ground-area basis, root-tip surface area on a ground-area basis, and the ratio of ECM to fine root on a weight basis, specific enzymatic activities of ECM (enzyme activity on surface-area basis) for each enzyme. Significant differences of specific enzymatic activities among the sites were also compared within each host genus. For evaluating the overall performance of ECM per site (as an integration of exploration capacity and specific enzyme activity), we calculated the ground-area based ECM enzymatic activity by multiplying ECM surface area on a ground-area basis with specific enzymatic activity on ECM-surface-area basis. Because the samples of ECM surface area and specific enzymatic activity did not correspond with each other, we simulated the possible variance of the ground-area based ECM enzymatic activity by applying all pairs between the surface area and enzymatic activity in each site. Linear regression was used to evaluate the contributions of surface area or specific enzyme activity as explanatory variables to ECM surface area enzymatic activity as a response variable. Although both response and explanatory variables were not independent; the purpose of this analysis was to figure out which (surface area or specific enzyme activity) better explained the ground-area based ECM enzymatic activity. Linear regression was conducted in logarithmic scale (with + 1 to include 0) to normalize the distribution. The enzymatic stoichiometry of ECM for C to P was evaluated by the ratio of BG (as C demand) to AP (as P demand), and that for N to P by the ratio of NAG + LAP (as N demand) to AP in each site.

To evaluate the relationship of ECM enzyme activities and their stoichiometry with environmental factors, we constructed a generalized linear mixed effects model (GLMM) for each specific enzyme activity as a response variable with resource availabilities of C, N and P as fixed effects by using lmer function in the R package “lme4”^45^. Here ANPP (above-ground net primary productivity) was used as an index of the potential of C supply from host tree to ECM fungi, and soil N mineralization rate and the pool size of soluble soil P were used as nutrient availabilities for ECM fungi. The environmental variables were cited from previous reports consucted in the same site as this study (Tables 1, 2)^5,29,36–38,46^. ANPP was calculated as the sum of the above-ground biomass increment and the above-ground litterfall^5^. We preliminarily checked if there were no correlation among environmental variables (*P* < 0.05). We standardized each environmental variable to calculate the standardized partial coefficients. The ID of individual tree was selected as the random intercept. Logarithmic transformation was applied to specific enzyme activities to normalize the distribution. The amount of variance explained by the fixed effects only and the combined fixed and random effects of the GLMM models were calculated as the marginal *R*^2^(*R*^2^_merginal_) and conditional *R*^2^(*R*^2^_conditional_) respectively, using the methods developed by Nakagawa and Schielzeth, (2013)^47^ (r.squaredGLMM function in “MuMIn” package). All statistical analyses were conducted using the R statistical program, version 3.4.0 (R Development Core Team 2017).

## Results

### Biomass and exploration capacity and specific enzymatic activity of ECM

Mean biomass, surface area, and the ratio of ECM to fine-root tended to be greater in montane forests than in lowland forests (Table 3). Especially, ECM biomass and ECM ratio to fine root were the greatest in the montane Tertiary sedimentary forest (17 T). ECM specific enzymatic activities on an ECM surface area basis exhibited different trends. Specific enzymatic activities were significantly higher in the montane ultrabasic forest (17U) among the sites for the following three enzymes; BG, NAG, and LAP (*P* < 0.05, Table 4). In the case of AP and PPO, the specific enzymatic activities were significantly higher in the montane ultrabasic (17U) and Quaternary sedimentary (17Q) sites than in the other sites (*P* < 0.05, Table 4).

**Table 3:** Biomass and surface area of ectomycorrhizal (ECM) tips, and ECM ratio to fine-root in study sites (± SE, n = 10).

Site	ECM biomass (g m^−2^)	ECM surface area (m^2^ECM m^−2^)	ECM ratio to fine-root (w/w)
07T	2.25 ± 0.66 b	0.344 ± 0.075 ab	0.0116 ± 0.0035 ab
07U	1.96 ± 0.80 b	0.226 ± 0.047 b	0.0072 ± 0.0030 b
17T	11.41 ± 3.72 a	0.401 ± 0.062 a	0.0376 ± 0.0152 a
17U	4.04 ± 0.63 ab	0.534 ± 0.103 ab	0.0080 ± 0.0013 b
17Q	3.16 ± 0.83 b	0.485 ± 0.076 ab	0.0105 ± 0.0032 ab

Different letters indicate significant differences among sites determined by Tukey’s HSD post hoc tests (*P* < 0.05). Abbreviations for sites: 07T, lowland forest on Tertiary sedimentary rocks; 07U, lowland forest on ultrabasic serpentine rocks, 17T, montane forest on Tertiary sedimentary rocks; 17U, montane forest on ultrabasic serpentine rocks; 17Q, montane forest on Quaternary sedimentary rocks.

**Table 4:** (a) Specific enzyme activities (mean ± SE) on an ectomycorrhizal-surface-area basis and (b) ground-area based ectomycorrhizal enzyme activities (15 cm depth) in the five study sites.

Site	β-glucosidase	N-acetylglucosaminidase	Leucine aminopeptidase	Acid phosphatase	Polyphenol oxidase
(a) Specific enzyme activity on an ECM surface area basis (pmol min^−1^ mm^−2^)					
07T	22.67 ± 8.54 b	27.80 ± 14.79 b	1.27 ± 0.98 b	43.25 ± 5.38 b	0.0588 ± 0.0142 b
07U	22.11 ± 5.76 b	31.26 ± 11.97 b	1.64 ± 0.77 b	34.75 ± 4.69 b	0.0976 ± 0.0087 b
17T	17.70 ± 2.44 b	24.90 ± 5.15 b	0.55 ± 0.16 b	26.14 ± 1.93 b	0.0870 ± 0.0096 b
17U	46.01 ± 5.91 a	116.63 ± 21.77 a	22.76 ± 6.37 a	83.43 ± 13.20 a	0.1297 ± 0.0241 a
17Q	24.03 ± 3.71 b	54.72 ± 9.08 b	0.73 ± 0.23 b	85.23 ± 13.36 a	0.1266 ± 0.0140 a
(b) Ground-area based ectomycorrhizal enzyme activities (μmol min^−1^ m^−2^)					
07T	7.81 ± 1.15 bc	9.58 ± 1.93 b	0.436 ± 0.126 b	14.90 ± 1.05 c	0.02025 ± 0.00210 c
07U	5.00 ± 0.52 c	7.07 ± 1.03 b	0.371 ± 0.065 b	7.87 ± 0.52 d	0.02209 ± 0.00120 c
17T	9.45 ± 0.59 b	13.30 ± 1.11 b	0.296 ± 0.033 b	13.96 ± 0.66 cd	0.04645 ± 0.00257 b
17U	22.30 ± 1.24 a	56.52 ± 4.08 a	11.032 ± 1.117 a	40.43 ± 2.58 a	0.06283 ± 0.00452 a
17Q	9.63 ± 0.59 b	21.94 ± 1.41 a	0.293 ± 0.033 b	34.17 ± 2.11 b	0.05075 ± 0.00246 a

Enzyme activities were measured under the ambient temperature and soil pH. See Table 3 for the abbreviations of the sites. See Table S3 for the sample size of each site. Different letters indicate significant differences among sites determined by Tukey’s HSD post hoc tests (*P* < 0.05).

### Ground-area based ECM enzymatic activity

**Figure 1:**
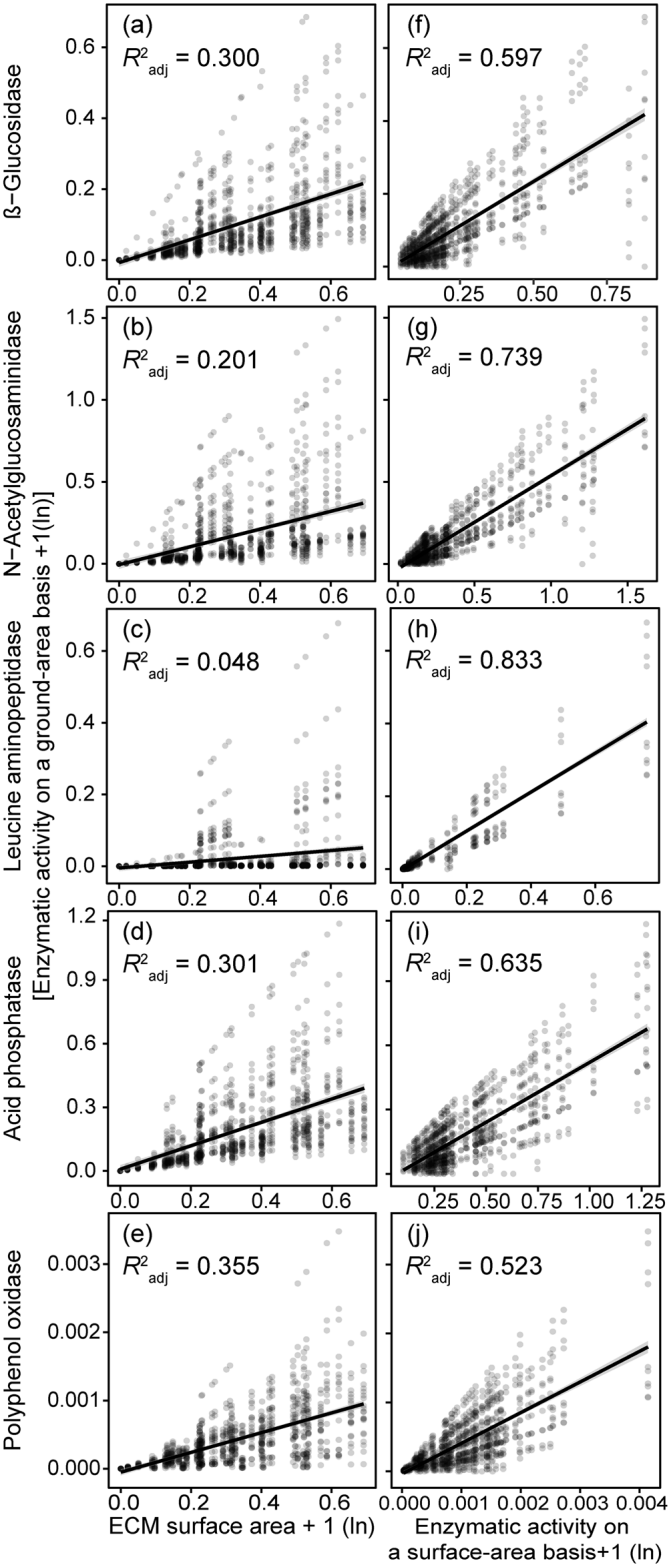
Linear regressions of ground-area based ectomycorrhizal enzyme activities with ectomycorrhizal (ECM) root tip surface area (**a**–**e**) and specific enzyme activities on an ECM-surface area basis (**f**–**j**) for five enzymes. Adjusted *R*^2^ was shown for each regression, *P* < 0.001 for all regressions.

The ground-area based ECM enzymatic activities were the highest in the 17U forest among the sites for BG, LAP, and AP (Table 4). In the case of NAG and PPO, the ground-area based enzymatic activities were significantly higher in the ultrabasic montane (17U) and Quaternary montane (17Q) sites than in the other sites (*P* < 0.05, Table 4). For all enzymes, the ground-area based enzymatic activities were better explained by the specific enzymatic activities rather than by the ground-area based ECM surface area as indicated by greater adjusted *R*^2^ (Fig. 1).

### Enzymatic stoichiometry

**Figure 2:**
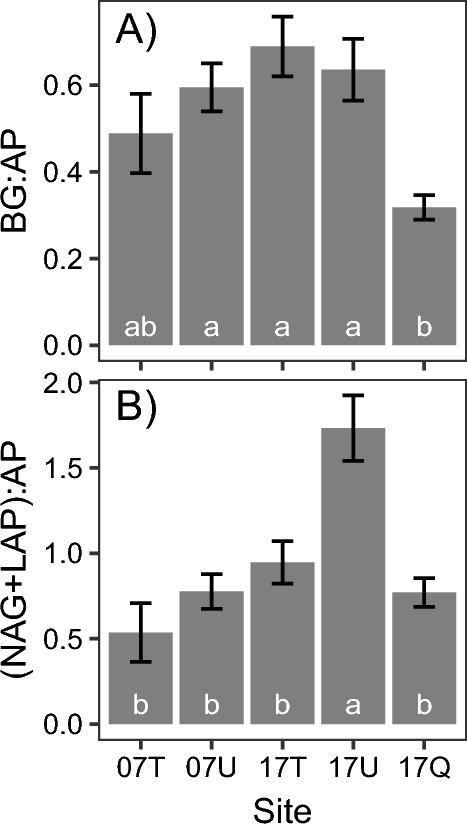
The stoichiometry of ECM enzymatic activity expressed as the ratio of BG (organic carbon degradation) to AP (organic phosphorus degradation) (diagram **A**), and NAG + LAP (organic nitrogen degradation) to AP (organic phosphorus) (diagram **B**). Different letters indicate significant differences between sites, determined by Tukey’s HSD post hoc tests. BG; β-glucosidase, NAG; N-acetylglucosaminidase, LAP; leucine aminopeptidase, AP; acid phosphatase. See Table 3 for the abbreviations of the sites. See Table S3 for the sample size of each site.

The stoichiometry of ECM enzymatic activity showed a different trend between the ratio of BG:AP and the ratio of (NAG + LAP):AP (Fig. 2). The BG:AP ratio was in a similar range among the sites except for Quaternary montane site (17Q), where show a significantly lower than the other site. The ratio of (NAG + LAP):AP was significantly higher only in the ultrabasic montane site (17U) than in the other sites, which is a different trend from the other enzymatic activities.

### Effect of elements on ECM enzymatic activities

**Figure 3:**
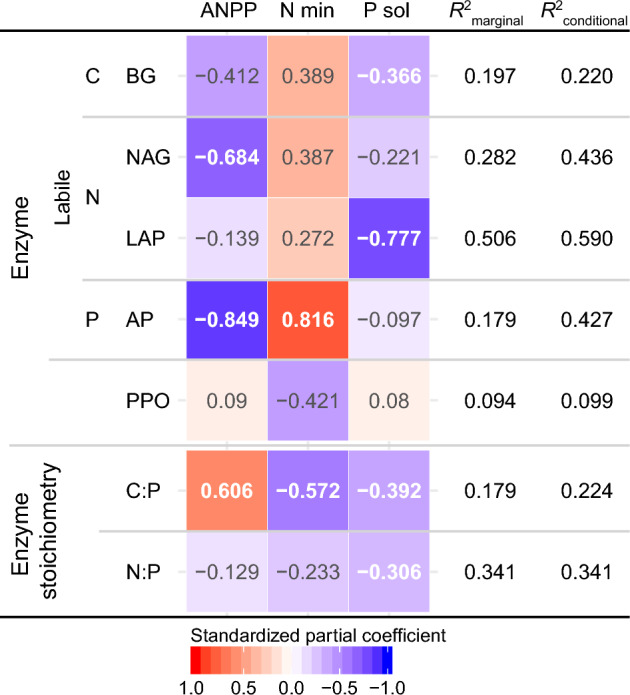
Summary of generalized linear mixed models (GLMMs) of each specific enzyme activity and enzyme stoichiometry with carbon (ANPP), nitrogen (N min) and phosphorus (P sol P) availabilities. White and gray values represent the significant (*P* < 0.05) and non-significant variables, respectively. *R*^2^_marginal_ and *R*^2^_conditional_ indicate the amount of variance explained by the fixed effects only and the combined fixed and random effects of GLMMs respectively. PPO; Polyphenol oxidase, for the acronyms of other enzymes, see Fig. 2. See Table S3 for the sample size applied from each site. ANPP; above-ground net primary productivity, N min; nitrogen mineralization rate, P sol; soluble phosphorus.

The results of GLMMs for each enzyme activity and enzymatic stoichiometry exhibited significant positive or negative effects of environmental factors (Fig. 3). ANPP negatively affects NAG and AP, and positively affects C:P. N mineralization rate of soil positively and negatively affects AP and C:P, respectively. The pool size of soluble P in soil negatively affects BG, LAP, C:P, and N:P. Particularly, GLMM of LAP represents the higher rate of variance explained by the fixed effects than the other enzymes (Fig. 3, R^2^_marginal_ = 0.506).

### Effect of host tree genera on ECM enzymatic activities

**Figure 4:**
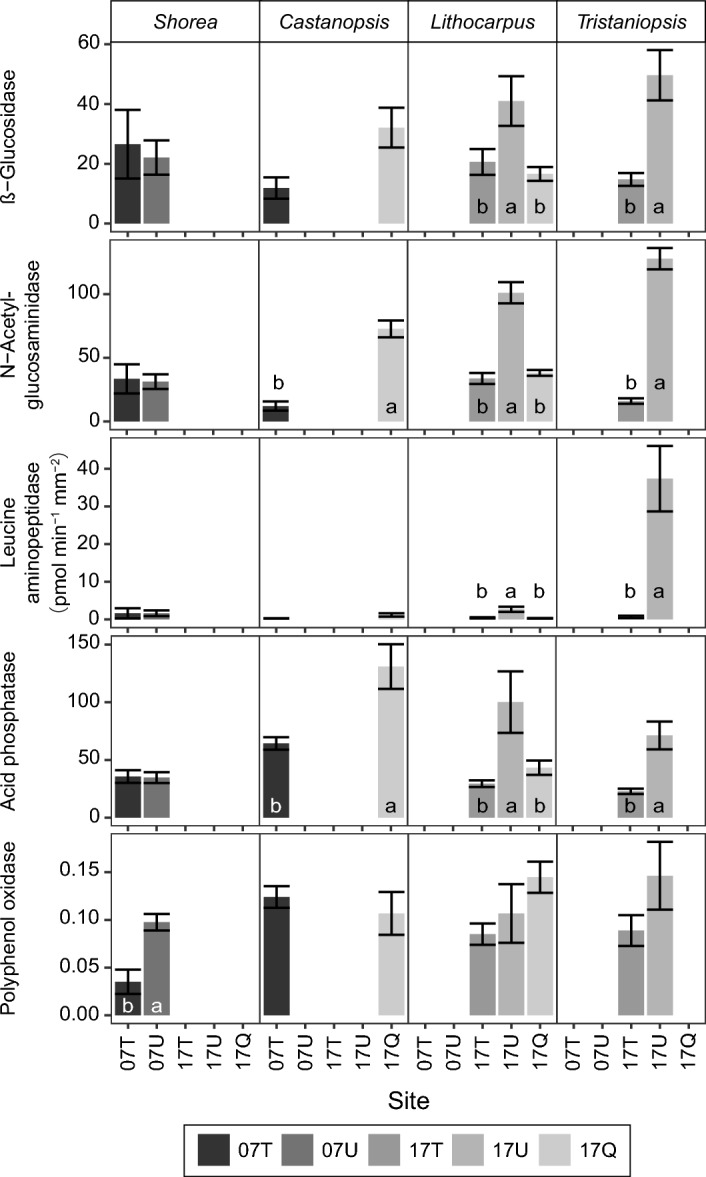
Ectomycorrhizal enzyme activities (mean ± SE) of host genera, *Shorea* (Dipterocarpaceae), *Castanopsis*, *Lithocarpus* (Fagaceae), and *Tristaniopsis* (Myrtaceae), measured under ambient conditions (same temperature and pH in sites). Different letters indicate significant differences between sites, determined by Tukey’s HSD post hoc tests. See Table 3 for the abbreviations of the sites. See Table S3 for the sample size of each site.

The comparison of enzymatic activity among the sites within the same host genus of ECM suggests similar patterns with the comparison that includes all the host genera described above (Fig. 4). ECM enzymatic activity of *Shorea* was similar between the two lowland sites except for polyphenol oxidase. In the case of *Castanopsis* distributed at both altitudes, enzymatic activity was relatively high in the montane forests although the difference was insignificant. The enzymatic activities of two dominant genera in the montane forests, *Lithocarpus* and *Tristaniopsis*, were significantly higher in the ultrabasic site than in the other sites for most enzymes.

## Discussion

In this study, we simultaneously investigated the two facets of exploration capacity and specific enzyme activity of ECM fungi, which has rarely been attempted previously. Our findings indicate that specific enzyme activities, rather than the root-tip surface area of ECM, better explain the variation in ground-area based enzymatic activities across study sites. (Fig. 1 and Table 4). We acknowledge potential impacts of multi-year sampling on our results. Yet, their reliability is supported by the moderate seasonal conditions in the tropical study sites. The nutrient acquisition capacity of ECM is primarily determined by its specific enzyme activity (i.e., enzymatic activity on ECM surface area basis) in tropical ecosystems. This is likely a response to carbon supply limitations from host trees in phosphorus-deficient sites as previously reported^5,48^. Limited C supply for ECM fungi at low ANPP site was suggested by the result that ECM biomass and its ratio to fine-root were lower in the ultrabasic site than in the sedimentary site in both montane and lowland forests (Table 3). Hence, enhancing enzymatic activities rather than expanding the surface area could be adaptive for ECM fungi to acquire nutrient efficiently under the C limitation in P deficient forests.

Specific enzymatic activities on an ECM-surface-area basis were associated with availabilities of C (as indexed by ANPP), N and P across our study sites (Fig. 3, Table 4). Because the ECM is mostly responsible for degradation and nutrient-releasing enzymes on root tips, exhibited by the higher enzymatic activities of ECM tip than those of non-ectomycorrhizal tips across all enzymes as indicated Table S4 and previously documented^17,26^. This suggests that ECM fungi are vital to biogeochemical cycles in tropical forest ecosystems, likely in response to the availability of C, N and P. Specific enzymatic activities were negatively affected by C (ANPP) and P availability (Fig. 3). Particularly, the activity of AP was negatively affected by C (ANPP) rather than by P availability. Contrary to our results, adaptive responses of fine-roots and the P-use efficiency of above-ground vegetation to P deficiency have been reported on Mount Kinabalu^5,7,49^. In the same site, Ushio et al.^50^ reported a significant increase of both specific root length and phosphatase activity of fine roots in the P deficient site. In addition, Kitayama^29^ found a significant negative correlation of specific root length and phosphatase activity of fine roots with the labile organic P content in soils, suggesting that labile organic P is an essential P resource for the trees and associated microbes^51^. Our results indicate that ECM fungi, however, do not respond to P deficiency directly by increasing P-releasing enzyme activity, suggesting an alternative mechanism of ECM fungi involving another nutrient enzyme under P deficiency.

Notably, the specific activities for the enzymes degrading not only organic P but also organic C (BG) and N (NAG and LAP) were high in the ultrabasic montane forest (17U) site where P was extremely deficient (Table 4). We suggest the following reasons for the simultaneous enhancement of C, N, and P enzyme activities on ECM. The increase of these enzyme activities might facilitate the P gain indirectly by degrading the carbon skeleton organic compound to release bound phosphorus^52^. Higher activity of recalcitrant C degrading enzyme (i.e., PPO) in the 17U site also supports this hypothesis. Moreover, it could be associated with the property of heterotrophy of ECM fungi. Generally, ECM fungi are considered to depend on the C supply from host plant as an exchange with another nutrient. Nevertheless, they have the latent saprotrophic capacity for carbon assimilation^53^, which was possibly stimulated under the insufficient carbon supply from host plant in this site. C allocation to mycorrhizal fungi could be diminished under P limitation because net primary production in both above- and below-ground systems is limited^5,48^. Indeed, P availability negatively affected the activity of the C degrading enzyme BG (Fig. 3). Such C limitation might enhance the latent saprotrophic ability (i.e. “facultative saprotrophy”^54,55^) of ECM fungi as an alternative source of C from host plant^56^. The concept of facultative saprotrophy on ECM fungi is not a mainstream^57^. Neverthless the specific ecophysiological roles of ECM fungi in P-deficient tropical forests remain underexplored. Emerging evidence of this study could supports “plan B” hypothesis by Talbot et al. (2008)^53^, suggesting increased enzyme production by ECM fungi when plant-derived nutrients are scarce^54,58^.

On the other hand, the enzymatic stoichiometry indicated a higher demand of ECM fungi for N when P is deficient, because (NAG + LAP): AP ratio (an index of N to P demand) and BG:AP (and index to C to P demand) were negatively affected by the pool size of soluble P (Fig. 3). This suggests that ECM fungi invest disproportionately more for enzymes to degrade N than to P with decreasing P availability. This is probably because N is an essential element of enzymes as with the other fundamental components of fungi such as non-enzyme protein, nucleic acid, and cell wall (chitin)^59^. The demand for N is inevitably involved in the synthesis of those enzymes for C and P, similar to nitrogen fixation, in that phosphatase requires N^60^. However, contrasting results have been reported in the meta-analysis of soil enzymatic activity in tropical forests^28^, in which BG:AP ratios were positively associated with soil P availability. The different response from our study might reflect specific nutrient acquisition strategies of target microorganisms in each study site. Soil enzymatic activities dealt by Waring et al., (2014)^28^ are mainly driven by free-living soil microbes including fungi and bacteria which are obligate saprotroph gaining C by organic matter degradation. By contrast, we investigated ECM fungi which mainly acquire C from host plants and have a facultative saprotrophic capacity. These differences between ECM fungi and free-living microbes might have resulted in the different specific responses of enzymatic stoichiometry.

Altitude was positively associated with ECM biomass and enzymatic activities, which were greater at montane sites than in lowland sites, even though sampling depth in this study was limited to the top 15 cm. However, enzymes typically activated at higher temperature, up to an optimum according to biological metabolism theory^61^. One reason may be related to the biogeographical patterns of ECM fungi that the ECM fungal richness is higher at mid-latitudes than in the equatorial tropical and boreal regions within the northern hemisphere^62^. Altitudinal distributions of ECM fungi on Mount Kinabalu suggest that the ECM fungal richness peaks in montane forests^39^. Likewise, ECM biomass may change with climate conditions. ECM biomass and its ratio to fine-root tend to be higher in cooler regions based on a European latitudinal experiment^63,64^. Such climate conditions are likely linked to the dominance of ECM fungi in relation to host productivity and nutrient availability.

The composition of ECM host trees also changes along altitude, but the composition of host trees does not seem to affect the community-wide ECM enzymatic activity as indicated by *Castanopsis* (Fagaceae) in our study. The enzymatic activity of *Castanopsis* was higher in the montane forest than in the lowland forest (Fig. 4). Dipterocarpaceae represents one of a few ectomycorrhizal taxa in the lowland forest and it has been believed that its ECM symbiosis with distinctive exploration capacities of soil nutrients is a reason for their successful domination in the lowland forest where the majority of trees are associated with arbuscular mycorrhiza^65^. However, our results indicate that specific enzymatic activities on *Shorea* ECM were not greater than those on the other host tree taxa (Fig. 4).

## Conclusion

ECM fungi enhance the specific enzyme activity (enzymatic activity on an ECM-surface area basis) rather than the exploration capacity (ECM root-tip surface area) to maintain the capacity of nutrient acquisition (i.e. the ground-area based ectomycorrhizal enzyme activities) in P deficient tropical forests. The less dependence of ECM fungi on the exploration capacity in these forests may be related to the limitation of C supply from host trees. These mechanisms of ECM fungi could contribute to maintaining the nutrient cycling and productivity of the tropical forest ecosystems under nutrient (particularly P) deficiency. Although some of the important aspects of ECM fungi such as the performance of soil exploring extraradical mycelium and the nutrient exchange between ECM fungi and host plants are still outstanding^11,17,56^, we have highlighted the importance of enzymatic activities of ECM, which reflects the nutrient acquisition strategies through ectomycorrhizal symbiosis in tropical rain forests.

### Supplementary Information

Supplementary Information 1.

Supplementary Information 2.

## Data Availability

The data-sets generated and analyzed during this study are included in this published article (and its supplementary information files).
