# Supplementary material for: Exploration capacity versus specific enzymatic activity of ectomycorrhizas in response to primary productivity and soil phosphorus availability in Bornean tropical rainforests
Source: Sci Rep. 2024 Feb 3;14:2842. doi: 10.1038/s41598-024-53234-6 (PMC10838334; doi:10.1038/s41598-024-53234-6)
Supplement: Supplementary file 2 — Supplementary Information 2. [file 41598_2024_53234_MOESM2_ESM.docx]

Supplementary materials

Title: Exploration capacity vs. specific enzymatic activity of ectomycorrhizas in response to primary productivity and soil phosphorus availability in Bornean tropical rainforests

Authros: Kei-ichi Okada^1, 2, 3^, Daiki Yokoyama^3, 4^, Shin-ichro Aiba^5, 6^ and Kanehiro Kitayama^3^

^1^Faculty of Bioindustry, Tokyo University of Agriculture, Abashiri, Japan

^2^ Graduate School of Environment and Information Sciences, Yokohama National University, Yokohama, Japan

^3^ Graduate school of Agriculture, Kyoto University, Kyoto, Japan

^4^ Center for Sustainable Resource Science, RIKEN, Yokohama, Japan

^5^Graduate School of Science and Engineering, Kagoshima University, Kagoshima Japan

^6^ Faculty of Environmental Earth Science, Hokkaido University, Sapporo, Japan

Corresponding author: Kei-ichi Okada (ko207453@nodai.ac.jp)

Figure S1

Anatomy of ectomycorrhizae observed on the root tips of each host genus distributed on Mount Kinabalu, Borneo. The ectomycorrhizal structure was not found on the root tips on genus *Leptospermum* (Myrtaceae).


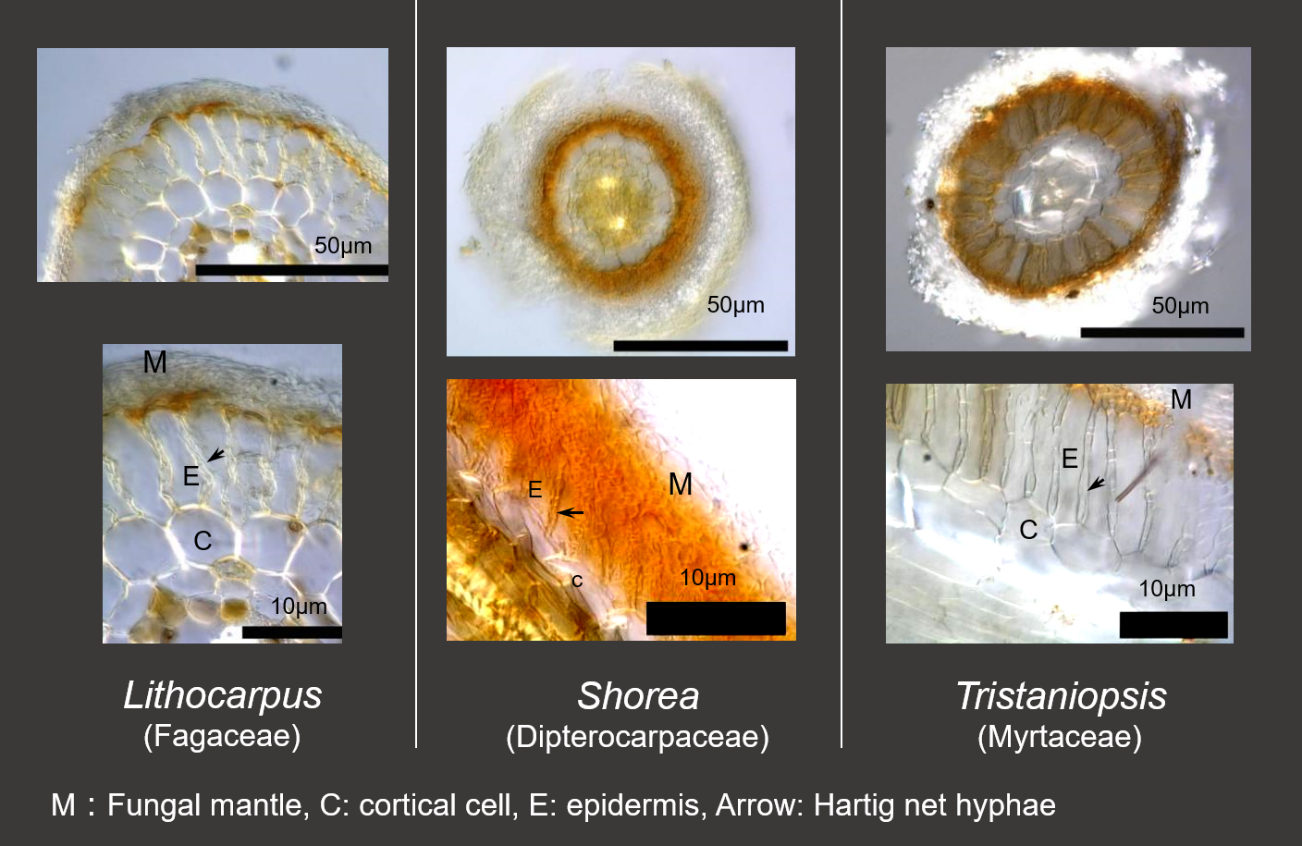


Table S1 Relative basal area (%) of ectomycorrhizal host genera in the five sites. Bold values in each site indicate the genera applied to the enzyme analyses. Only genus *Tristaniopsis* was recognized as an ectomycorrhizal host tree in Myrtaceae in the study sites (see Materials and Methods). For the abbreviations of the sites, see Fig. 1.

| Family of ectomycorrhizal host trees | 07T | 07U | 17Q | 17T | 17U |
| --- | --- | --- | --- | --- | --- |
| Dipterocarpaceae | **22.7** | **36.9** |  | 0.1 |  |
| Fagaceae | **6.9** | 1.6 | **21.1** | **15.2** | **5.6** |
| Myrtaceae (*Tristaniopsis*) | 3.0 |  | 0.3 | **11.2** | **31.5** |
| Total | 32.6 | 38.5 | 21.4 | 26.5 | 37.1 |

Table S2 list of target host tree species for ectomycorrhizal root sampling

| Study sites |  | Family | Species |
| --- | --- | --- | --- |
|  |  |  |  |
| Lowland | Tertiary | Dipterocarpaceae | *Shorea argentifolia* |
| (700 m) |  | Fagaceae | *Castanopsis javanica* |
|  | Ultrabasic | Dipterocarpaceae | *Shorea laevis* |
| Montane | Quaternary | Fagaceae | *Castanopsis* sp. |
| (1700 m) |  | Fagaceae | *Lithocarpus lampadalius* |
|  | Tertiary | Fagaceae | *Lithocarpus clementianus* |
|  |  | Myrtaceae | *Tristaniopsis clementis* |
|  | Ultrabasic | Fagaceae | *Lithocarpus rigidus* |
|  |  | Myrtaceae | *Tristaniopsis kinabaluensis* |

Table S3 The number of sampling trees (replicates) for enzyme activity assay of ectomycorrhizas in the study sites. Dip.:Dipterocarpaceae, Fag. :Fagaceae, Myrt. : Myrtaceae, *Shor.* : *Shorea*, *Cast*. : *Castanopsis*. *Litho*. : *Lithocarpus*, *Tris*. : *Tristaniopsis*

| Study sites |  | Dip. | Fag. | | Myrt. | Total |
| --- | --- | --- | --- | --- | --- | --- |
|  |  | *Shor* | *Cast.* | *Litho.* | *Tris.* |  |
| Lowland | Tertiary | 3 (11) | 1 (4) |  |  | 4(15) |
| (700 m) | Ultrabasic | 7 (20) |  |  |  | 7(20) |
| Montane | Quaternary |  | 3 (11) | 4 (12) |  | 7(23) |
| (1700 m) | Tertiary |  |  | 4 (11) | 4 (11) | 8(22) |
|  | Ultrabasic |  |  | 4 (8) | 4(11) | 8(19) |

Table S4 Enzymatic activities of non-ectomycorrhizal (NM) tips and a comparison with that of ectomycorrhizal (ECM) tips by Welch's t-test. For the acronyms of enzymes, see Fig. 4.

|  | BG | | | NAG | | | LAP | | | AP | | | PPO | | |
| --- | --- | --- | --- | --- | --- | --- | --- | --- | --- | --- | --- | --- | --- | --- | --- |
|  | (pmol min^−1^ mm^−2^) | | | | | | | | | | | | | | |
| ECM | 26.07 | ± | 24.54 | 50.72 | ± | 66.01 | 5.13 | ± | 14.79 | 54.86 | ± | 48.43 | 0.1013 | ± | 0.0698 |
| NM | 7.62 | ± | 4.60 | 11.18 | ± | 10.47 | 0.71 | ± | 2.05 | 20.14 | ± | 19.37 | 0.0091 | ± | 0.0256 |
| *P* | < 0.001 | | | < 0.001 | | | 0.004 | | | < 0.001 | | | < 0.001 | | |
